# Optimizing Backbone Networks Through Hybrid–Modal Fusion: A New Strategy for Waste Classification

**DOI:** 10.3390/s25103241

**Published:** 2025-05-21

**Authors:** Houkui Zhou, Qifeng Ding, Chang Chen, Qinqin Liao, Qun Wang, Huimin Yu, Haoji Hu, Guangqun Zhang, Junguo Hu, Tao He

**Affiliations:** 1 College of Mathematics and Computer Science, Zhejiang A & F University, Hangzhou 311300, China; zhouhk@zju.edu.cn (H.Z.); dqf@stu.zafu.edu.cn (Q.D.); cc@stu.zafu.edu.cn (C.C.); lqqnn@stu.zafu.edu.cn (Q.L.); gloria@zafu.edu.cn (G.Z.); hujunguo@zafu.edu.cn (J.H.); 2 Zhejiang Provincial Key Laboratory of Forestry Intelligent Monitoring and Information Technology, Hangzhou 311300, China; 3 College of Information Science and Technology, Zhejiang Shuren University, Hangzhou 311300, China; 4 College of Information Science and Electronic Engineering, Zhejiang University, Hangzhou 310027, China; yhm2005@zju.edu.cn (H.Y.); haoji_hu@zju.edu.cn (H.H.); 5 State Key Laboratory of CAD & CG, Hangzhou 310027, China

**Keywords:** image recognition, waste classification, transformer, model fusion, optimization strategy

## Abstract

**Highlights:**

**What are the main findings?**

- A novel multi-modal fusion model HFWC-Net (Hybrid–Modal Fusion Waste Classification Network) for efficient waste classification is proposed.

**What is the implication of the main finding?**

- HFWC-Net effectively reduces the amount of calculation and training time while maintaining high performance.
- HFWC-Net can improve the accuracy of automatic garbage sorting and protect the natural environment.

**Abstract:**

With rapid urbanization, effective waste classification is a critical challenge. Traditional manual methods are time-consuming, labor-intensive, costly, and error-prone, resulting in reduced accuracy. Deep learning has revolutionized this field. Convolutional neural networks such as VGG and ResNet have dramatically improved automated sorting efficiency, and Transformer architectures like the Swin Transformer have further enhanced performance and adaptability in complex sorting scenarios. However, these approaches still struggle in complex environments and with diverse waste types, often suffering from limited recognition accuracy, poor generalization, or prohibitive computational demands. To overcome these challenges, we propose an efficient hybrid-modal fusion method, the Hybrid-modal Fusion Waste Classification Network (HFWC-Net), for precise waste image classification. HFWC-Net leverages a Transformer-based hierarchical architecture that integrates CNNs and Transformers, enhancing feature capture and fusion across varied image types for superior scalability and flexibility. By incorporating advanced techniques such as the Agent Attention mechanism and the LionBatch optimization strategy, HFWC-Net not only improves classification accuracy but also significantly reduces classification time. Comparative experimental results on the public datasets Garbage Classification, TrashNet, and our self-built MixTrash dataset demonstrate that HFWC-Net achieves Top-1 accuracy rates of 98.89%, 96.88%, and 94.35%, respectively. These findings indicate that HFWC-Net attains the highest accuracy among current methods, offering significant advantages in accelerating classification efficiency and supporting automated waste management applications.

## 1. Introduction

With the acceleration of urbanization and rapid population growth, the improvement of residents’ living standards has led to a diversification of consumption structures and a sharp increase in domestic waste. It is expected that global solid waste generation will reach 2.2 billion tons per year by 2025 [1]. Effectively managing this growing amount of waste has become a significant challenge. While waste sorting and recycling are effective methods for dealing with urban waste and protecting the environment, the wide variety and shapes of waste require substantial human investment for accurate classification [2]. Additionally, residents’ insufficient awareness of classification and incomplete implementation of relevant policies result in unsatisfactory waste classification outcomes [3]. Accurate waste classification technology can effectively distinguish different types of waste, significantly improve the feasibility of harmless treatment, ensure hazardous waste is specially treated, and reduce threats to the environment. Automated waste processing technology, leveraging artificial intelligence and machine learning, further enhances sorting efficiency and reduces operating costs [4,5]. Therefore, developing effective automatic waste classification methods has significant academic and practical importance.

Manual waste classification poses significant challenges due to its reliance on human labor, which is inherently time-consuming, costly, and prone to errors [6]. Workers are required to visually inspect and categorize heterogeneous waste materials—including plastics, metals, and organics—often under suboptimal conditions. This process is not only inefficient but also subject to inconsistencies arising from human fatigue and subjectivity, leading to frequent misclassification. For example, non-recyclable plastics may be incorrectly sorted as recyclable, thereby compromising the effectiveness of downstream recycling operations and increasing processing costs. The variability in waste appearance—caused by contamination, deformation, or degradation—further complicates accurate sorting, reducing throughput and diminishing resource recovery rates. These limitations place a growing burden on waste management systems, especially in the context of escalating urban waste volumes. Moreover, improper sorting of hazardous materials, such as batteries, can result in significant environmental risks, including soil and water contamination. In light of these challenges, there is an urgent need for robust, automated classification solutions. Leveraging the capabilities of deep learning, such systems promise substantial improvements in accuracy, efficiency, scalability, and overall cost-effectiveness, making them a critical advancement for modern waste management [7].

Previous studies have applied deep learning to waste classification with promising results. In 2016, Yang et al. from Stanford University introduced the TrashNet dataset, comprising 2527 images, which has become a cornerstone for developing waste classification models [8]. Subsequent research has leveraged transfer learning and innovative architectures to enhance performance. For example, Zhang et al. applied DenseNet169 to their NWNU-TRASH dataset, outperforming other algorithms despite high computational costs [9]. Wu et al. improved the VGG architecture for better feature extraction, though it struggled with varying object scales and interpretability [10]. Similarly, Lin et al. developed MSWNet using ResNet50 for urban waste sorting, boosting efficiency via transfer learning but requiring substantial resources [11]. Hossen et al. proposed GCDN-Net, enhancing interpretability with activation mapping (Score-CAM), yet it demands extensive labeled data and processing power [12]. Despite these advancements, limitations such as limited sample diversity, insufficient accuracy, and high computational demands persist. To address these, Transformer models have emerged as a promising alternative. Hu et al.’s Swin Transformer improved accuracy and efficiency but faced challenges with imbalanced datasets and complex environments [13]. Alrayes et al. achieved 95.8% accuracy on TrashNet using an enhanced Transformer, though it too required significant resources [14]. For resource-constrained settings, lightweight models have been explored. Xia et al.’s YOLO-MTG offers robust multi-target detection under varying lighting, albeit with slower inference on some hardware [15]. Gupta et al.’s SmartBin, built on InceptionNet and Raspberry Pi, enables real-time classification but still demands considerable power [16]. While these methods have propelled waste classification forward, their practical deployment is often hindered by resource demands, scalability, and adaptability. Thus, selecting and tailoring models to specific application needs is critical for achieving optimal results. Existing studies have combined image classification technology and machine learning methods to improve waste classification based on image recognition, but they still have certain limitations. These limitations include (1) low diversity of waste image samples, far from actual samples; (2) insufficient accuracy of waste classification models to meet actual needs; (3) deficiencies in real-time response and deployment, hindering timely and effective waste classification; and (4) limited generalization ability, restricting applicability in different scenarios.

To overcome the limitations of existing automated classification systems, this study introduces an efficient hybrid–modal fusion approach named HFWC-Net. ‘Hybrid–modal fusion’ refers to the integration of multiple image features through a hybrid architecture that combines convolutional neural networks (CNNs) and Transformer models, enhancing the model’s ability to perceive complex waste types. Unlike traditional multi-scale fusion, which focuses on extracting features at different spatial scales, or multimodal fusion, which emphasizes integrating information from distinct data sources (e.g., images and text), hybrid–modal fusion achieves finer-grained feature integration at the architectural level. Specifically, HFWC-Net leverages CNNs for local feature extraction and Transformers for global feature perception, employing a layered design and an Agent Attention mechanism to effectively fuse diverse image characteristics. This hybrid structure not only improves adaptability to complex backgrounds and varied waste types but also enhances computational efficiency and training speed through the LionBatch optimization strategy. By incorporating this approach, HFWC-Net addresses key challenges in waste classification, offering a robust solution for modern waste management.

The main contributions of this study include (1) constructing a new dataset, MixTrash, containing 135 categories of waste, with highly diverse image data meeting real-world classification needs; (2) integrating a new attention mechanism, Agent Attention, into the backbone model to effectively manage computing resources and focus on relevant input data, improving model performance with high expressiveness and low computational complexity; (3) proposing a new optimization strategy, LionBatch, combining model pruning technology with a new optimizer to reduce computational requirements and improve operational efficiency, thereby reducing resource consumption while maintaining high accuracy.

## 2. Materials and Methods

### 2.1. Self-Built Dataset MixTrash

**Figure 1:**
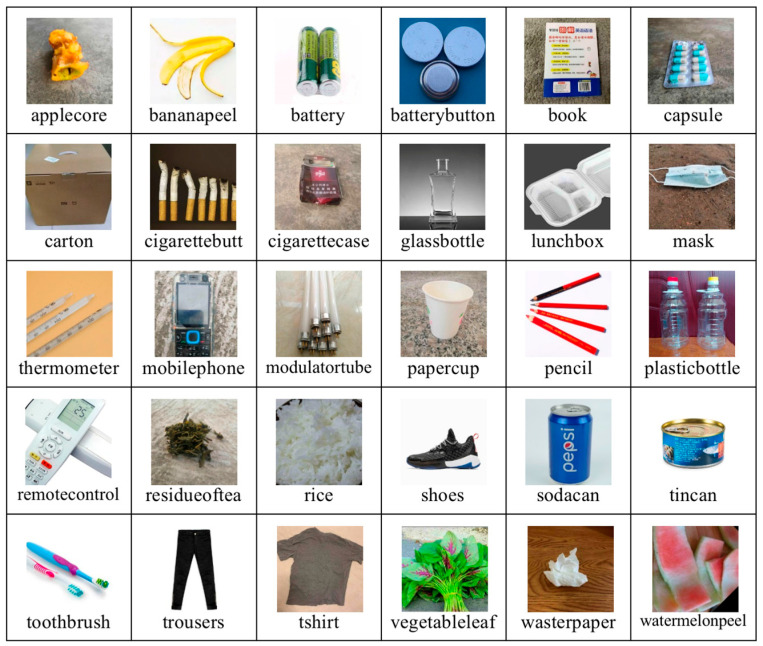
Illustration of representative images from the MixTrash dataset.

Currently, there are few public datasets available in the field of waste identification, with most related studies relying on the TrashNet dataset [8]. The TrashNet dataset is a small collection of recyclable waste images, including six categories: glass, paper, cardboard, plastic, metal, and general waste, comprising a total of 2527 photos. However, this dataset has several shortcomings: the sample size is too small; the distribution of different types of waste is uneven; the background of the images is too uniform, which does not reflect real-world conditions and hinders the generalization ability of trained models. To address these limitations, we constructed a new waste image dataset, MixTrash, using Internet searches and manual photography. The MixTrash dataset includes 135 different categories of waste, such as banana peels, old toothbrushes, cans, old clothes, wastepaper, leftovers, and waste batteries, totaling 52,324 images (Figure 1). The images in MixTrash feature varied backgrounds, and the number of images for different types of waste is balanced, ensuring high data diversity that better meets the needs of real-world scenarios.

The MixTrash dataset is a multi-category image dataset specifically designed for waste recognition tasks, comprising four primary waste categories—recyclables, kitchen waste, hazardous waste, and other waste—encompassing a total of 135 subclasses (Table 1). The dataset is developed based on a standardized waste classification system: recyclables include 74 subclasses such as metal tools (e.g., anvils, scissors), plastic products (e.g., shampoo bottles, plastic bottles), wooden items (e.g., chair, wooden shovels), textiles (e.g., old clothes), and paper products (e.g., books, paper cups); kitchen waste comprises 21 subclasses covering easily perishable organic matter (e.g., fruit cores, peels) and processed food residues (e.g., sausages, biscuits); hazardous waste includes 15 subclasses such as toxic metal items (e.g., thermometers, button batteries) and pure electronic waste (e.g., power banks, circuit boards); and other waste features 26 subclasses like contaminated composite materials (e.g., wet wipes, old gloves). All images were captured in real-world settings (e.g., kitchens, streets) under varied lighting conditions and object variations. Compared to existing datasets such as TrashNet, MixTrash provides notable enhancements in sample scale, inter-class balance, and environmental diversity, offering essential data support for the development of efficient and robust waste classification models.

### 2.2. HFWC-Net

The Transformer architecture, with its self-attention mechanism, can effectively capture global features by focusing on the relationships between various regions in an image. Traditional convolutional neural networks (CNNs) primarily process information through local receptive fields, which can limit their ability to capture broader contextual information, resulting in inferior performance in complex image analysis tasks. Additionally, the hierarchical structure of CNNs often requires more layers to expand the receptive field, leading to larger, harder-to-optimize models. To address these shortcomings, the Transformer architecture is not only better suited for complex image analysis tasks, but its design also naturally supports parallel computing. This makes Transformers more efficient in processing large-scale datasets and accelerating the training process. To further enhance performance, this study proposes a new network architecture, HFWC-Net. HFWC-Net is designed to combine the efficient self-attention mechanism of the CSWin Transformer with deep feature processing capabilities specifically tailored for complex images. This approach leverages the CSWin Transformer’s strengths in multi-scale and multi-dimensional information processing. HFWC-Net addresses the limitations of CNN models in image processing through fine-grained feature fusion and efficient optimization strategies, thereby improving the model’s overall performance and adaptability.

HFWC-Net’s hybrid–modal fusion framework effectively integrates the strengths of both CNNs and Transformers into a unified architecture. This fusion is achieved through a hybrid design that combines the local feature extraction capabilities of CNNs with the global context modeling power of Transformers. Specifically, the network first employs a convolutional token embedding layer to capture fine-grained local features from input waste images, such as edges and textures. This is followed by a sequence of CSWin blocks and HFWC blocks, which leverage cross-shaped Window Attention and Agent Attention mechanisms to model long-range dependencies and multi-scale contextual relationships. Merge Blocks play a crucial role in progressively reducing spatial dimensions while increasing channel depth, thereby enabling seamless integration of local and global features within the network’s four-stage hierarchical structure. This hybrid fusion strategy offers several advantages: by incorporating the global perceptual ability of Transformers, it overcomes the limited receptive field of CNNs; and through a coarse-to-fine integration scheme, it enriches feature diversity while reducing computational overhead. These characteristics make HFWC-Net a highly practical and efficient solution for intelligent waste classification tasks.

**Figure 2:**
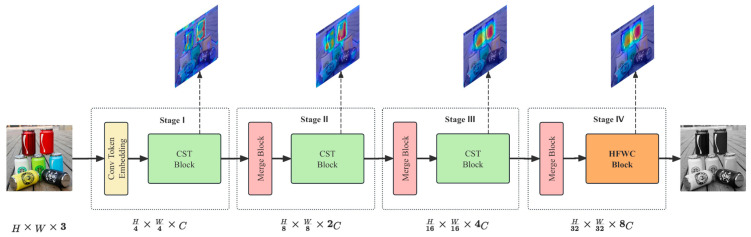
The overall architecture of our proposed HFWC-Net.

The overall architecture of HFWC-Net is shown in the image (Figure 2). This neural network utilizes a four-layer pyramid structure to efficiently process and abstract image features. Each layer’s design goals and structures are carefully configured to optimize feature extraction and representation capabilities. The first three layers of the network form the basic feature extraction and processing modules, each comprising multiple CSWin Blocks. These blocks employ the self-attention mechanism of a cross-shaped window to process image data, which is particularly suited for capturing local features and their spatial correlations. In this four-layer pyramid structure, the spatial resolution of the image is gradually reduced through the fusion module, Merge Block, while the channel dimension is doubled at each upward layer. This design allows the network to progressively enhance the abstraction ability of features while maintaining a low computational cost. The Merge Block is primarily responsible for reducing the number of tokens and merging features from adjacent layers, enabling the model to capture more comprehensive and high-level features as the number of layers increases.

Through this pyramid setting, the first three layers are primarily responsible for feature extraction, ranging from basic to relatively complex. The CSWin Block in each layer refines and strengthens the features at different scales, preparing them for advanced feature integration. The final layer utilizes the HFWC Block, which replaces the traditional cross-shaped window self-attention mechanism with the Agent Attention mechanism. This effectively reduces the number of features involved in the calculation. The introduction of agent tokens allows the attention calculation to focus on significant features, avoiding redundant computations of the full-size feature matrix. This achieves linear complexity and significantly improves the model’s computational efficiency. By optimizing the processing flow and reducing the computational burden, the HFWC Block not only enhances the model’s capability to handle complex data but also improves its ability to capture details and distinguish different categories. Through innovative structural design, HFWC-Net effectively improves the depth and breadth of feature processing, enhances the model’s expressiveness, and optimizes computational efficiency. This enables HFWC-Net to more accurately understand objects in complex categories, making it particularly effective for challenging tasks such as waste classification.

### 2.3. Cswin Transformer Block (CST Block)

The CSWin Transformer is an innovative Transformer architecture designed for computer vision tasks, proposed by Microsoft Research Asia in 2021 [17]. Through its novel self-attention mechanism and position encoding method, the CSWin Transformer optimizes the theoretical applicability of the Transformer architecture and demonstrates significant performance advantages in actual vision tasks.

**Figure 3:**
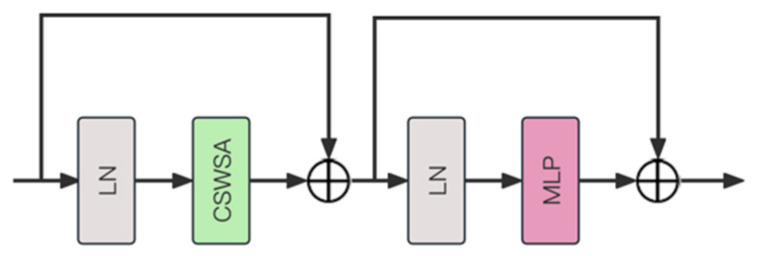
The architecture of CST block.

The structure of the CSWin Transformer Block is shown in the image (Figure 3). Layer normalization (LN) is used at the beginning and middle of the module to normalize the mean and variance of the input layer [18]. Between the two layer normalization stages, the cross-shaped window self-attention mechanism (CSWSA) is employed. CSWSA allows the model to focus on key regions of the image while maintaining computational efficiency by forming a cross-shaped window on the input image or feature map. Following CSWSA is a multilayer perceptron (MLP), which typically consists of several fully connected layers and uses nonlinear activation functions such as ReLU between these layers [19]. The MLP further processes the output of the self-attention layer, increasing the nonlinearity of the network and enabling the model to learn more complex feature representations.

**Figure 4:**
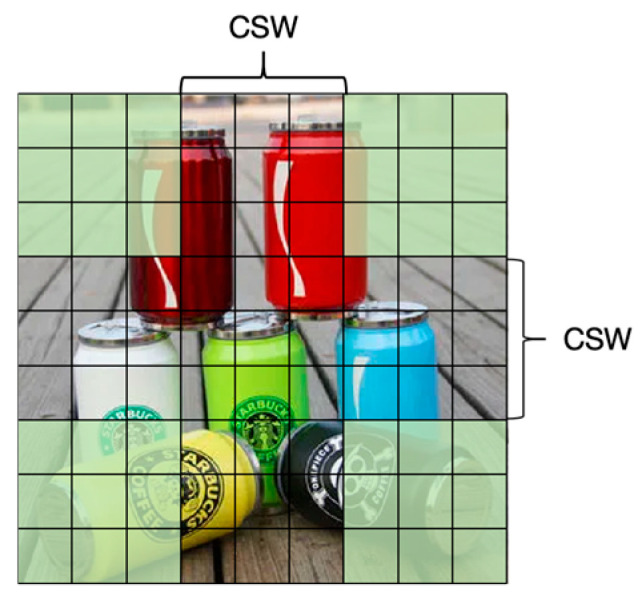
Cross-Shaped Window Self-Attention.

One of the core features of the CSWin Transformer is its cross-shaped window self-attention mechanism. Unlike traditional Transformers that apply self-attention across the entire image or within fixed-size windows, CSWSA first splits the input image into multiple small windows. Each window is further divided into a cross center and four corners, with the center window forming a cross shape covering strips in the horizontal and vertical directions (Figure 4). Within each cross-shaped window, the model calculates self-attention independently, meaning it evaluates the relationship of each patch within the window with all other pixels in the same window. This design allows the model to more effectively capture long-range dependencies in the image while reducing computational complexity.

### 2.4. Merge Block

**Figure 5:**
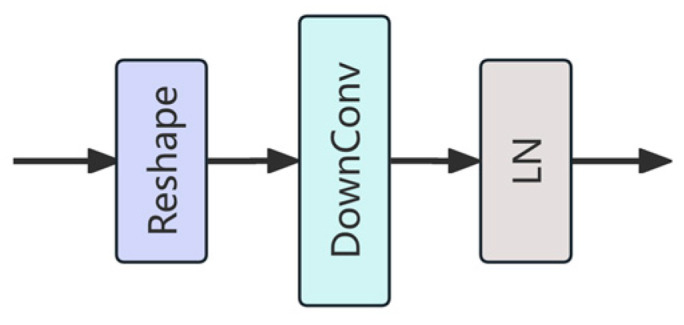
The architecture of Merge block.

Merge Block is a dedicated module in this study designed to enhance feature fusion by enabling efficient integration and transmission of multi-level feature information. The module comprises three core stages: reshaping, convolution-based downsampling, and normalization (Figure 5). First, the input sequence of tokens is reshaped to reconstruct its spatial structure, enabling the model to reestablish spatial correlations among features. This transformation is essential for bridging the gap between sequence-based representations and spatial operations. Next, a 3 × 3 convolution with a stride of 2 (denoted as “DownConv”) is applied to simultaneously reduce spatial resolution and expand the channel dimension, thereby enriching the semantic content while reducing computational cost. Finally, LN is applied to stabilize the feature distribution and facilitate more robust training dynamics. Unlike traditional vision Transformer modules—such as ViT’s fixed Patch Merging or Swin Transformer’s window-based downsampling—the Merge Block leverages learnable convolutional operations, allowing it to adaptively capture local spatial dependencies and contextual information during the fusion process. Its CNN-based design not only enhances local detail modeling but also ensures higher computational efficiency and better compatibility with modern hardware. Within the HFWC-Net framework, the Merge Block serves as a crucial inter-stage connector, promoting seamless and effective feature propagation across hierarchical layers.

### 2.5. HFWC Block

**Figure 6:**
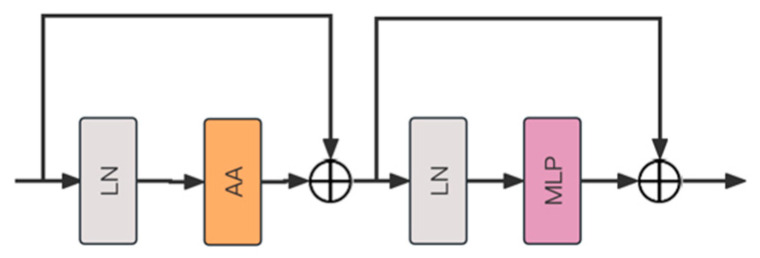
The architecture of HFWC Block.

HFWC Block features an advanced Agent Attention Mechanism (AA) to optimize information processing flow (Figure 6). The module employs layer normalization (LN) at both the initial and middle stages to standardize the mean and variance of the input layer. Between these two layer normalization stages, the Agent Attention Mechanism is integrated. This mechanism optimizes attention calculation by introducing agent tokens, which reduces the complexity of direct calculations and improves the efficiency and accuracy of information processing. Following the Agent Attention Mechanism, the module also incorporates a multilayer perceptron (MLP), which consists of several fully connected layers and embeds nonlinear activation functions between layers. The HFWC Block with Agent Attention excels in computational efficiency and fast processing, making it suitable for various application scenarios.

Agent Attention was proposed by Han et al. to balance computational efficiency and representation capabilities [20]. In visual Transformers, the traditional global attention mechanism has high expressive ability but also high computational complexity, limiting its application in high-resolution scenes. The cross-attention mechanism expands the attention area of each token within the Transformer block by performing self-attention in horizontal and vertical strips. However, processing high-resolution images and large-scale data can still pose a high computational burden. Agent Attention combines the advantages of Softmax [21] and linear attention [22]. It introduces agent tokens, simplifies the aggregation process of global information, maintains high expressive ability, and reduces computational complexity, thus overcoming the performance bottlenecks encountered by the cross-attention mechanism in processing large-scale data. This attention mechanism can more effectively focus on key areas of the image, thereby improving classification accuracy and efficiency, which is particularly advantageous for processing large amounts of data and complex tasks.

**Figure 7:**
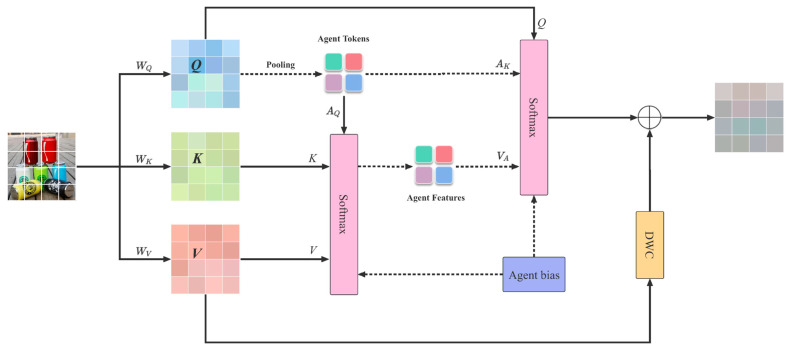
Agent Attention.

The Agent Attention mechanism inserts an additional set of token A into the attention triplet $\left(Q,K,V\right)$ calculation to form a new quadruple attention paradigm $\left(Q,K,V,A\right)$. The Agent Attention mechanism consists of two traditional Softmax operations (Figure 7). The first Softmax is applied to the triplet $\left(A,K,V\right)$, where token A is used as a query to aggregate information from the value V to form a new agent value $V_{A}$. The attention matrix is between A and K, effectively reducing the need to directly process all data and greatly reducing computational complexity. The second Softmax is calculated on the triple $\left(Q,A,V_{A}\right)$, where $V_{A}$ is the result of the first step. The newly introduced token A acts as a “proxy” for query Q, as they directly collect information from K and V and then pass the results to Q. Query token Q no longer needs to communicate directly with the original key K and value V. This feature reduces the quadratic complexity of Softmax to linear complexity, while retaining the global context modeling capability.

The algorithm of the Agent Attention mechanism is as follows. First, we simplify Softmax and linear attention to

(1) $$\begin{array}{c} O^{S}=Softmax\left(QK^{T}\right)V≜{Attn}^{S}\left(Q,K,V\right)\\ O^{\phi }=\phi \left(Q\right)\phi {\left(K\right)}^{T}V≜{Attn}^{\phi }\left(Q,K,V\right) \end{array}$$

where Q,K,V represent query, key, and value matrices, and Agent Attention can be written as

(2) $$O^{A}={Attn}^{S}\left(Q,A,{Attn}^{S}\left(A,K,V\right)\right)$$

equivalent to

(3) $$\begin{array}{ll} O^{A} & =Softmax\left(QA^{T}\right)Softmax\left(AK^{T}\right)V\\ & =\phi_{q}\left(Q\right)\phi_{k}{\left(K\right)}^{T}V\\ & ={Attn}^{\phi_{q}/k}\left(Q,K,V\right)\;\;\; \end{array}$$

The Agent Attention mechanism consists of two Softmax operations, responsible for agent aggregation and agent broadcasting, respectively. Specifically, the agent token A is first regarded as a query, and attention calculation is performed between A, K, and V to aggregate the agent feature $V_{A}$ from all values. Subsequently, A is used as the key and $V_{A}$ as the value in the second attention calculation of the query matrix Q to broadcast the global information of the agent feature to each query token, obtaining the final output O. The newly defined agent token A essentially acts as an agent of Q, aggregating global information from K and V, and then broadcasting it back to Q, maintaining the global context modeling capability. To better utilize the position information, the Agent Attention adds carefully designed agent biases $B_{1}$, $B_{2}$:

(4) $$O^{A}=Softmax\left(QA^{T}+B_{2}\right)Softmax\left(AK^{T}+B_{1}\right)V$$

Based on these designs, the Agent Attention mechanism can be expressed as

(5) $$O^{A}=Softmax\left(QA^{T}+B_{2}\right)Softmax\left(AK^{T}+B_{1}\right)V+DWC\left(V\right)$$

Modern Transformer models typically use sparse attention [23] or Window Attention [17] to reduce the computational burden of Softmax attention. Benefiting from linear complexity, these attention mechanisms enjoy the advantages of large and even global receptive fields while ensuring manageable computational costs. Agent Attention integrates these two forms of attention, drawing on their strengths to balance computational efficiency and expressiveness. By introducing agent tokens, relevant information, Agent Attention effectively focuses the model’s capacity on important features within the input data.

### 2.6. New Optimization Strategy LionBatch

AdamW has become a cornerstone optimizer in deep learning due to its decoupled weight decay mechanism, which separates regularization from gradient updates to enhance training stability [24]. However, despite its effectiveness, AdamW has some significant drawbacks, particularly in terms of memory consumption and computational efficiency. AdamW stores multiple moving averages of gradients and squared gradients for each parameter, which can quickly become resource-intensive, especially in models with numerous parameters. This architecture leads to inefficient GPU memory utilization and elevated computational costs during backpropagation. Furthermore, AdamW exhibits pronounced sensitivity to hyperparameter configurations (e.g., learning rate, $\beta_{1}$ momentum coefficients, weight decay coefficient), necessitating meticulous tuning to avoid suboptimal convergence or training instability. To address these limitations, Lion optimizer introduces a resource-efficient paradigm by redefining momentum utilization [25]. Unlike AdamW’s adaptive learning rate mechanism, Lion employs a sign-based update rule that retains only a single momentum buffer, reducing memory consumption while eliminating redundant gradient magnitude calculations. This design enables linear computational scaling with model size, enhancing training throughput for deep architectures. Crucially, Lion replaces AdamW’s complex hyperparameter dependencies with a simplified two-parameter framework (learning rate, $\beta_{1}$ momentum), demonstrating superior robustness across tasks. This simplicity allows Lion to converge more quickly and efficiently, especially on large datasets, without the need for fine-tuning complex learning rates or momentum parameters. This makes Lion more accessible and effective for researchers, especially when dealing with complex models and large datasets that require both speed and accuracy.

Traditional pruning techniques reduce model size and computational complexity by removing some weights or neurons in the network, including unimportant weight pruning and random pruning [26]. While traditional pruning helps improve model running speed and reduce storage requirements, it often leads to a decline in model performance, especially when the pruning ratio is high, as the model may lose key information. To address the shortcomings of traditional pruning techniques, Qin et al. from the National University of Singapore proposed an unbiased dynamic data pruning method, InfoBatch [27]. Traditional pruning methods often introduce biases in gradient expectations by discarding important samples that help the model generalize. InfoBatch avoids this bias by adjusting the gradient weights of the remaining samples while pruning them (called gradient rescaling), maintaining the same gradient expectations as the unpruned dataset.

**Figure 8:**
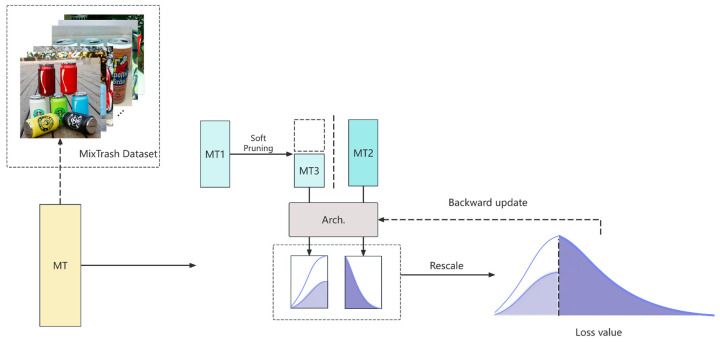
Illustration of the LionBatch framework.

To better optimize the model, this paper proposes a new optimization strategy, LionBatch, which combines the Lion optimizer and InfoBatch pruning. The main algorithm flow is shown in the image and table (Figure 8 and Algorithm 1). By integrating these two technologies, our new optimization strategy not only improves the training speed and resource utilization efficiency of the model but also significantly reduces the computing resources required during the training process while maintaining high classification accuracy. LionBatch tracks momentum during model training and computes updates through symbolic operations, thereby reducing memory overhead and ensuring update magnitudes are consistent across all dimensions. LionBatch implements dynamic pruning, allowing pruning decisions to be adjusted based on the real-time performance of the data during training. This allows for more detailed control of the training process. Through this dynamic and unbiased pruning method, LionBatch can reduce training costs while maintaining model performance, achieving lossless training acceleration. These innovative designs demonstrate significant advantages in improving model efficiency and effectiveness.

The detailed steps of the LionBatch algorithm are as follows.
**Algorithm 1.** LionBatch Optimization Strategy$given\;\beta_{1},\beta_{2},\lambda ,\eta ,f,r$ $\mathrm{initialize}\;\theta_{0},m_{0},z_{0},p_{0}\leftarrow 0$ $\mathrm{while}\;\theta_{t}$ not converged do update sample scores based on loss ${\;p}_{t}\leftarrow \int_{z}Loss\left(z_{t},\theta_{t}\right)\rho \left(z_{t}\right)dz_{t}$ ${\;\theta }_{t}\leftarrow arg\;{min}_{\theta }\frac{1}{r}p_{t}$ ${\;g}_{t}\leftarrow \nabla_{\theta }f\left(\theta_{t-1}\right)$ update model parameters $c_{t}\leftarrow \beta_{1}m_{t-1}+\left(1-\beta_{1}\right)g_{t}$ ${\;\theta }_{t}\leftarrow \theta_{t-1}-\eta_{t}($sign$\left(c_{t}\right)+\lambda \theta_{t}-1)$ $\mathrm{update}\;\mathrm{EMA}\;\mathrm{of}\;g_{t}$ ${\;m}_{t}\leftarrow \beta_{2}m_{t-1}+\left(1-\beta_{2}\right)g_{t}$ end while $\mathrm{return}\;\theta_{t}$


First, calculate the average loss of the entire dataset and directly prune the part with smaller loss. After each epoch or several epochs of training, recalculate the average loss of the entire dataset. For pruned and untrained samples, use the previous loss; for pruned samples, use the updated loss after training. For data samples p with losses less than the average loss, perform pruning with a certain probability r. Due to the reduction in the number of samples, the gradient g of the entire dataset will change, leading to inconsistency with the expected gradient of the original dataset. To address this, rescale the gradient g of the data samples with losses less than the average loss. Then, use the Lion optimizer to optimize the pruned data subset, and calculate the current update momentum c using the gradient g and the previous momentum m. Use the sign function to specify the update direction to update the model parameter θ. Combine the previous momentum m and the current gradient to update the exponential moving average (EMA). Finally, repeat the above process to gradually optimize the model parameters until the desired performance indicators are achieved.

The LionBatch optimization strategy significantly reduces training time and computing resource consumption while maintaining high classification accuracy. By employing dynamic learning rates and momentum terms, LionBatch enables the model to converge to the global optimal solution faster, reducing the number of training iterations. This strategy not only makes the optimization process more efficient and stable but also reduces the risk of over-fitting and under-fitting. Additionally, LionBatch optimizes the model structure by dynamically removing redundant neurons, avoiding unnecessary computational burden and ensuring the model’s accuracy. The LionBatch strategy excels in optimizing computing resource consumption, which is particularly important for resource-constrained environments such as embedded or mobile devices, making it feasible to deploy deep learning models on these platforms.

### 2.7. Experimental Environment Set-Up

After building the HFWC-Net model, we used the PyTorch deep learning framework to train it in graphics processing unit (GPU) mode. In this experiment, two computer devices were used for the entire training and validation process. The detailed characteristics of the two devices are shown in the tables (Table 2 and Table 3). Machine 1 is primarily used to train CNN and Transformer architecture networks due to its strong computing power and efficient video memory management. This capability allows Machine 1 to handle a large number of matrix operations and data processing effectively. Machine 2, on the other hand, is used to run VMamba, as VMamba is more compatible with the Linux system, making its training on this device more efficient and convenient.

### 2.8. Experimental Evaluation Metrics

In this study, we used various deep learning models to conduct experiments on multiple datasets. To properly evaluate the performance of these models, we adopted established evaluation metrics, including Top1-Accuracy (Top1-Acc), precision, recall, and F1 score. These metrics are computed using data extracted from the confusion matrix, which contains key parameters such as the total number of test samples (ATs), true positives (TP), true negatives (TN), false positives (FP), and false negatives (FN). All values are calculated using the global confusion matrix, which consists of the results from cross-validation [28].

(6) $$\;Top1-Acc=\frac{\sum \left(TPs\right)}{ATs}\;$$

(7) $$\;Precision=\frac{TP}{TP+FP}$$

(8) $$\;Recall=\frac{TP}{TP+FN}$$

(9) $$\;F1-score=\frac{2TP}{2TP+FP+FN}$$

## 3. Results

### 3.1. Comparative Experimental Results

To evaluate the performance of the model proposed in this paper, we conducted a series of comparative experiments on the public datasets TrashNet [8] and the self-built waste dataset MixTrash. We selected numerous advanced classification models as references, including VGGNet [10], GoogleNet [16], ResNet [11], DenseNet [9], MobileNet [29], EfficientNet [30], ConvNext [31], ViT [32], DeiT [33], Swin Transformer [13], TNT [34], PiT [35], CaiT [36], BiFormer [37], and VMamba [38]. The experiment used the same loss function, learning strategy, and image preprocessing method to ensure the fairness of the results. The experimental results indicate that the improved model proposed in this study performs exceptionally well across the three datasets. The following sections present the results of the comparative experiments.

#### 3.1.1. Comparison Results of Garbage Classification Dataset

The results show the Top1-Acc, precision, recall, F1 score, and number of parameters for different models on the Garbage Classification dataset (Table 4). The experimental results reveal that traditional CNN models such as VGGNet, GoogleNet, ResNet, and DenseNet achieve similar accuracy, with Top1-Acc ranging from 93% to 94%. Among them, VGGNet has the highest Top1-Acc at 93.97%, but it also has a much larger number of parameters compared to the other three traditional CNN models. In contrast, lightweight models like MobileNet and EfficientNet achieve higher accuracy with fewer parameters by optimizing computational efficiency, with EfficientNet’s Top1-Acc being 2.74% higher than VGGNet’s. Transformer architecture models, including ViT, DeiT, Swin Transformer, CSwin Transformer, TNT, PiT, CaiT, and BiFormer, perform well, with Top1-Acc ranging from 95% to 97%, exceeding that of traditional CNN models by more than 2%. Among these, CSwin Transformer stands out by introducing cross-shaped Window Attention to effectively capture spatial patterns, achieving a Top-1 accuracy of 97.03%. This makes CSwin a strong baseline among vision Transformers, balancing accuracy and model complexity. Additionally, ConvNext combines the advantages of traditional CNN and Transformer models, achieving a Top1-Acc of 96.74%, though its parameter count far exceeds that of most CNN and Transformer models. VMamba also demonstrated efficient performance, achieving a Top1-Acc of 98.45%, second only to the HFWC-Net proposed in this study. The HFWC-Net proposed in this study achieved a Top1-Acc of 98.89% on the Garbage Classification dataset, significantly higher than traditional CNN models and current mainstream Transformer models. Particularly in terms of parameter volume, HFWC-Net offers similar accuracy to VMamba but with significantly fewer parameters, translating to lower operating costs and faster processing speeds in actual deployment. Moreover, HFWC-Net’s accuracy is 2.77% higher than that of the Swin Transformer, which performs well within the Transformer architecture, while its parameter volume is about 13% lower than that of the Swin Transformer. This reduction is particularly important in resource-constrained application scenarios. The outstanding performance of HFWC-Net is attributed to its innovative network architecture and optimization strategies, which greatly enhance the model’s learning efficiency and generalization ability. These results fully demonstrate the significant advantages of HFWC-Net in handling Garbage Classification tasks.

**Figure 9:**
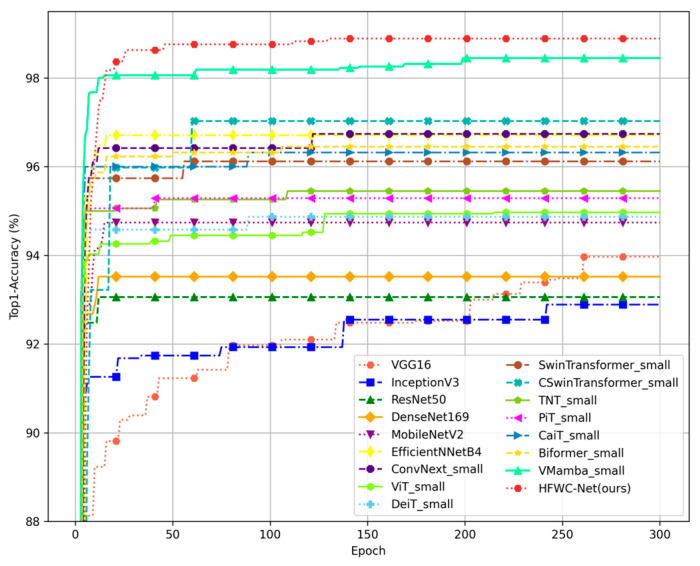
Training curve performance of the proposed method and other methods on the Garbage Classification.

The image details the trend of Top1-Acc of different models during training on the Garbage Classification dataset as the number of training rounds changes (Figure 9). It can be observed from the figure that the accuracy of most models increases rapidly within the first 50 epochs, with the accuracy of each model tending to stabilize as it approaches 300 epochs. Notably, some traditional CNN models, such as VGGNet and ResNet, exhibit slow convergence speeds during training. Specifically, the accuracy of these models still fluctuates significantly around 100 epochs, which may be related to their deeper network structures. In contrast, lightweight models such as MobileNet and EfficientNet show faster convergence speeds due to their optimized structures. This indicates that these models can efficiently complete training tasks under limited resources. Regarding Transformer models, such as Swin Transformer, CSwin Transformer, and BiFormer, they not only converge faster than traditional CNNs throughout the training cycle but also demonstrate higher accuracy in the early stages compared to traditional CNNs and lightweight models. This highlights the superior capability of Transformer models in handling classification tasks. It is worth mentioning that the HFWC-Net model achieved high accuracy early in the training process, second only to VMamba. Around 50 epochs, the accuracy of the HFWC-Net model surpassed VMamba, fully validating the efficiency and effectiveness of our proposed method.

#### 3.1.2. Comparison Results of TrashNet Dataset

The results show the Top1-Acc, precision, recall, F1 score, and number of parameters for different models on the TrashNet dataset (Table 5). The experimental results reveal significant differences in accuracy among traditional CNN models such as VGGNet, GoogleNet, ResNet, and DenseNet. Among these, GoogleNet has the lowest Top1-Acc at 83.50%. In contrast, lightweight models such as MobileNet and EfficientNet exhibit good performance. Notably, EfficientNet’s performance is comparable to traditional heavy models like VGGNet, but with only 13% of VGGNet’s parameters. Transformer architecture models, including ViT, TNT, CSwin Transformer, and BiFormer, perform well, with Top1-Acc rates above 90%, significantly higher than traditional CNN models. Among them, the baseline CSwin Transformer demonstrates particularly strong performance, outperforming most other Transformer variants. Its balanced accuracy and training stability underscore the effectiveness of the cross-shaped attention mechanism in capturing both local and global features, making it especially well-suited for complex classification tasks such as waste categorization. VMamba exhibited even better performance, with a Top1-Acc of 95.83%, 3.98% higher than ConvNext, further proving the advantages of these emerging models in handling complex classification tasks. The HFWC-Net proposed in this study performed the best among all models, achieving a Top1-Acc of 96.43% on the TrashNet dataset, significantly higher than all models except VMamba, demonstrating its substantial improvement in accuracy.

**Figure 10:**
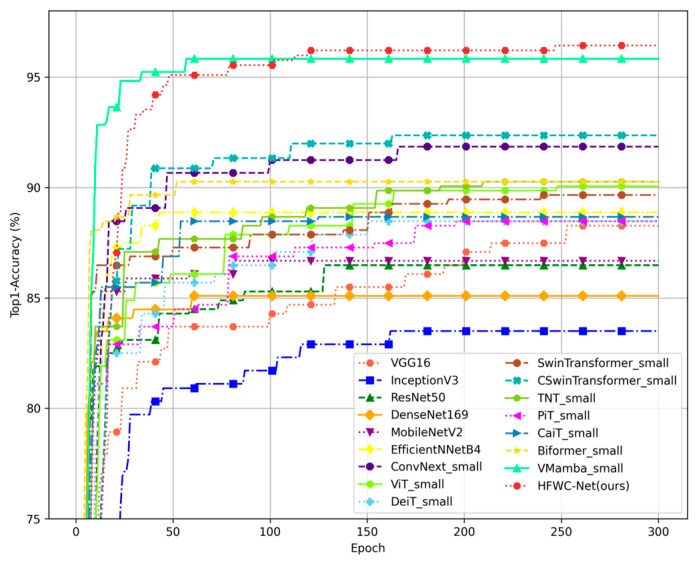
Training curve performance of the proposed method and other methods on the TrashNet.

The image details the Top1-Acc trends of different models as they train on the TrashNet dataset (Figure 10). It can be observed that the accuracy of most models fluctuates greatly in the early stages of training, stabilizing only after approximately 300 training cycles. Traditional CNN models such as VGGNet, ResNet50, DenseNet169, and GoogleNet improve slowly in the early stages, converge relatively slowly, and ultimately achieve lower final accuracy compared to newer models. Lightweight models like MobileNet and EfficientNet show faster convergence speeds, but their accuracy is only slightly higher than VGGNet, the best-performing traditional model, by about 0.6%. Among the Transformer models, ViT, TNT, and BiFormer perform well in both convergence speed and final accuracy, demonstrating the advantages of the Transformer architecture in processing image classification tasks. It is particularly noteworthy that HFWC-Net and VMamba improve rapidly in accuracy in the early stages of training, far exceeding all other models. HFWC-Net eventually surpasses VMamba at around 120 training cycles, indicating the efficiency of its optimization strategy and network architecture. These results highlight the design advantages of HFWC-Net in achieving high accuracy, making it ideal for handling complex image classification problems.

#### 3.1.3. Comparison Results of MixTrash Dataset

The results show the Top1-Acc, precision, recall, F1 score, and parameter count of different models on our self-built dataset MixTrash (Table 6). The experimental results indicate that traditional CNN models such as VGGNet, GoogleNet, ResNet, and DenseNet perform poorly. The Top1-Acc of VGGNet is only 72.11%, reflecting its inefficiency in processing complex datasets due to its large number of parameters and deep network structure. Among lightweight models, EfficientNet excels. Utilizing a compound scaling method, EfficientNet optimizes in multiple dimensions and achieves excellent performance with relatively few parameters. The overall performance of Transformer architecture models is relatively average, with Top1-Acc between 83% and 86%, due to the scale and complexity of the MixTrash dataset limiting the potential of the Transformer models. BiFormer performs relatively well, with a Top1-Acc of 87.28%. Its unique bidirectional feature extraction mechanism provides advantages in processing complex image tasks, but its overall performance still lags behind the emerging efficient models HFWC-Net and VMamba. CSwin Transformer, introduced as the baseline model, delivers highly competitive performance on MixTrash. With a Top1-Acc of 92.50%, it surpasses most Transformer variants, demonstrating stable convergence and strong generalization. VMamba achieved a Top1-Acc of 93.89% on the MixTrash dataset, demonstrating extremely high classification performance. However, its training time per epoch is the highest among all models, suggesting a significant computational cost despite its excellent accuracy. The HFWC-Net proposed in this study performed best among all models, achieving a Top1-Acc of 94.35% on the MixTrash dataset. Compared to other models, HFWC-Net not only has an advantage in accuracy but also achieves higher optimization efficiency regarding parameter quantity. For instance, compared to the Swin Transformer with a similar parameter count, HFWC-Net reduces the number of parameters by 11.72% while increasing accuracy by 9.03%. This outstanding performance fully demonstrates the great potential and advantages of HFWC-Net in processing complex data features and achieving efficient classification.

**Figure 11:**
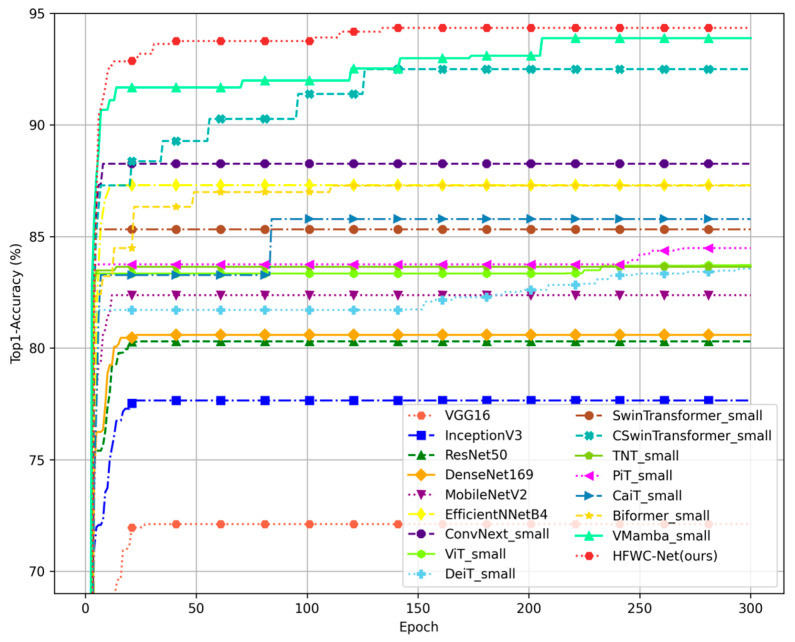
Training curve performance of the proposed method and other methods on the MixTrash.

**Figure 12:**
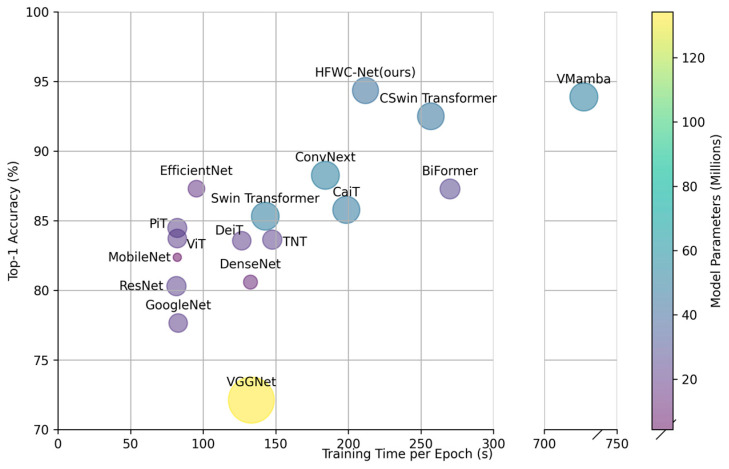
Scatter plot of MixTrash dataset result comparison.

The images show the relationship between the Top1-Acc, training time, and model parameter count of different models on the MixTrash dataset (Figure 11 and Figure 12). The scatter plot intuitively illustrates the performance and training efficiency of each model. Among traditional CNN models, VGGNet exhibits the lowest Top1-Acc, the longest training time, and the largest number of parameters. This indicates that VGGNet has a complex structure and low efficiency, making it difficult to handle complex datasets. Although GoogleNet, ResNet, and DenseNet have shorter training times, their Top1-Acc is relatively low. Lightweight models such as MobileNet and EfficientNet have shorter training times and higher Top1-Acc compared to most traditional CNN models. Particularly, EfficientNet achieves an accuracy of 87.30%, demonstrating excellent classification performance while maintaining a low number of parameters and short training time. Transformer architecture models such as ViT, DeiT, TNT, and PiT have accuracy rates between 83% and 86%, with training times slightly longer than those of lightweight models but shorter than those of traditional CNN models. While these models improve upon traditional CNNs, they do not surpass lightweight models. Swin Transformer and CaiT achieve high accuracy but have relatively long training times and large parameter counts, highlighting both their advantages and disadvantages on complex datasets. BiFormer performs relatively well, with a moderate training time and parameter count, showing certain advantages. CSwin Transformer, used as a baseline model in this study, demonstrates outstanding performance as well. Although its training time is longer than some lightweight models, it significantly outperforms most other Transformer variants, reflecting a strong balance between performance and computational cost. VMamba achieves a Top1-Acc close to the highest, but its training time is the longest, about five times that of mainstream Transformer models. This indicates that VMamba has outstanding classification performance but low training efficiency. HFWC-Net performs exceptionally well, standing out with the highest Top1-Acc and moderate training time. Under similar parameter conditions, HFWC-Net’s training time is significantly lower than VMamba’s, being only about one- third of it, and its accuracy is 6.09% higher than ConvNext. This demonstrates HFWC-Net’s excellent classification performance and efficient training efficiency. Overall, HFWC-Net exhibits the best overall performance across various indicators, fully demonstrating its powerful capability in waste classification tasks.

### 3.2. Ablation Experiment Results

In addition, on the MixTrash dataset, we conducted ablation experiments to verify the effectiveness of each improvement strategy. The experimental results clearly show that each improvement strategy, including the introduction of a new attention mechanism and optimized training algorithm technology, significantly enhances the model’s classification performance. This study uses CSwin Transformer as the baseline network for this experiment. First, the self-attention mechanism of the last layer of the CSwin Block was replaced with a parallel Agent Attention mechanism to prevent loss caused by incomplete feature extraction. The improved model benefits from the Transformer’s powerful global feature extraction capability, with an accuracy increase of 1.03% compared to the backbone network (Table 7). To accelerate network training, we introduced the LionBatch optimization strategy, which significantly reduced training time while maintaining high classification accuracy. The experimental results indicate that Model F, utilizing the LionBatch strategy, shortened the training time and reduced the computational load, despite having similar parameter counts and FLOPs. Ultimately, the proposed HFWC-Net model achieved an accuracy of 94.35% on the MixTrash dataset, an increase of 1.85% compared to the original model. Additionally, the training time was reduced from 24.67 h to 24.22 h. Therefore, HFWC-Net can quickly and accurately classify waste in actual scenarios, meeting the needs of real-time waste classification.

**Figure 13:**
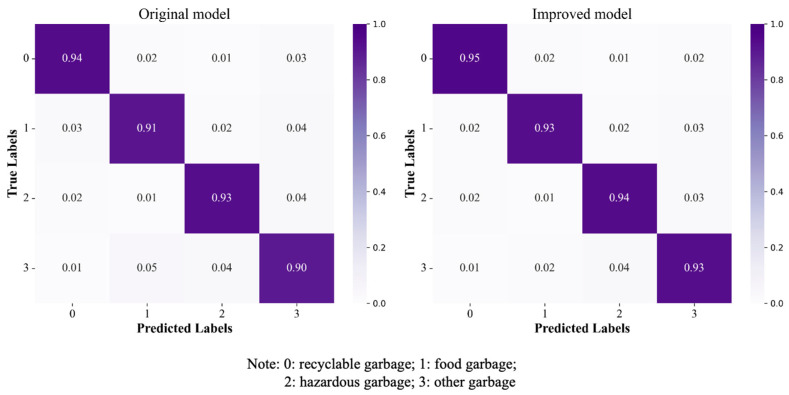
Confusion matrix.

To verify the performance improvement of the HFWC-Net model compared to the original model, we used the test set to evaluate the classification effects of both models. According to the number of correctly predicted samples in the confusion matrix, the improved model increased the identification accuracy of recyclable waste, kitchen waste, hazardous waste, and other waste by 1%, 2%, 1%, and 3%, respectively (Figure 13). This performance improvement indicates that HFWC-Net can more accurately identify and classify different types of garbage in classification tasks. The confusion matrix of the original model reveals that the main reason for the lower accuracy is the confusion between kitchen waste and other garbage during the classification process. This issue may arise from similarities in appearance and characteristics between some food waste and other waste, causing difficulty for the model in distinguishing these categories. Specifically, kitchen waste and other garbage may share similarities in color, shape, and texture, making it challenging for the original model to accurately differentiate them. HFWC-Net significantly enhances the model’s feature extraction capabilities and classification performance by introducing innovative technologies such as multi-level feature fusion and new optimization strategies. These improvements enable HFWC-Net to better capture subtle differences in garbage images and improve the ability to identify complex features. Overall, the improvement of the HFWC-Net model not only increases the overall classification accuracy but also demonstrates the effectiveness and superiority of HFWC-Net in Garbage Classification tasks. This provides a more reliable solution for practical applications.

### 3.3. Real-World Performance Metrics

To assess the real-world applicability of HFWC-Net for waste classification, we evaluated its inference efficiency on the MixTrash dataset using Machine 1 (as detailed in Table 2). The experiment focused on single-image inference to simulate practical scenarios, such as real-time waste sorting in automated systems. The table presents the GPU memory consumption and average inference time for HFWC-Net alongside several baseline models, including ResNet, EfficientNet, ConvNeXt, ViT, Swin Transformer, CSwin Transformer, and BiFormer (Table 8). HFWC-Net demonstrates a balanced performance, consuming 266.2 MB of GPU memory and achieving an average inference time of 7.9 ms per image. Compared to lightweight models like EfficientNet, HFWC-Net uses slightly more memory but maintains competitive inference speed. In contrast, models like ConvNeXt and Swin Transformer exhibit significantly higher memory usage, making them less suitable for resource-constrained environments despite faster or comparable inference times. ViT offers the fastest inference but sacrifices accuracy on complex datasets like MixTrash, as shown in prior results. CSwin Transformer performs similarly to HFWC-Net in memory usage but is slightly slower, while BiFormer is the least efficient, with the longest inference time and higher memory demand. These results highlight HFWC-Net’s efficiency, making it well-suited for real-time waste classification on edge devices while maintaining high accuracy.

## 4. Discussion

### 4.1. Interpreting the Superior Performance of HFWC-Net in Waste Classification

The experimental results underscore the remarkable efficacy of the HFWC-Net in addressing the challenges of waste image classification, achieving Top-1 accuracy rates of 98.89%, 96.43%, and 94.35% on the Garbage Classification, TrashNet, and MixTrash datasets, respectively. These figures not only highlight HFWC-Net’s exceptional performance but also validate the core hypothesis driving this study: that a Transformer-based architecture, augmented by hybrid–modal fusion, the Agent Attention mechanism, and the LionBatch optimization strategy, can substantially outperform traditional convolutional neural networks (CNNs) and existing Transformer models in both accuracy and processing efficiency. This success is particularly evident when considering the diversity and complexity of the datasets tested. For instance, the Garbage Classification dataset, with its broad range of waste categories, benefits from HFWC-Net’s ability to integrate multi-modal features, achieving a near-perfect 98.89% accuracy. Similarly, on TrashNet—a smaller, less diverse dataset with 2527 images across six categories—HFWC-Net’s 96.43% accuracy demonstrates its adaptability to varying dataset scales, surpassing previous benchmarks like Alrayes et al.’s 95.8% [14]. The self-built MixTrash dataset, with its 135 categories and 52,324 images reflecting real-world variability, further showcases HFWC-Net’s robustness, achieving 94.35% accuracy despite the increased complexity and sample diversity.

This superior performance can be attributed to the synergistic design of HFWC-Net’s components. The Transformer-based backbone, leveraging the CSWin Transformer’s cross-shaped window self-attention, excels at capturing global and local feature relationships, overcoming the limitations of CNNs’ localized receptive fields. The hybrid–modal fusion approach enhances this capability by integrating diverse image attributes—such as shapes, colors, and textures—into a cohesive feature representation, as evidenced by the model’s consistent high precision, recall, and F1 scores across all datasets (e.g., 98.82% F1 score on Garbage Classification). The Agent Attention mechanism further refines this process by focusing computational resources on salient features, reducing redundancy and boosting efficiency, a critical factor in achieving linear complexity over traditional quadratic attention models. Meanwhile, the LionBatch optimization strategy accelerates training and reduces resource demands, as seen in the ablation study (Table 7), where its inclusion cuts training time from 24.67 to 24.22 h while maintaining accuracy gains. Collectively, these innovations enable HFWC-Net to process large-scale, heterogeneous waste data swiftly and accurately, aligning with the study’s aim to develop a scalable solution for automated waste classification.

### 4.2. Limitations and Future Directions of HFWC-Net

Despite its advantages, the proposed waste classification method HFWC-Net still presents several limitations. First, the model’s feature extraction capabilities may be insufficient when processing low-resolution garbage images or images with missing fine details, which can lead to reduced classification accuracy. This issue becomes particularly prominent in complex real-world scenes captured by cameras, where factors such as lighting variations, occlusions, and motion blur can significantly degrade model performance. Additionally, for anomalous data or garbage categories that are underrepresented or absent in the training set, HFWC-Net may exhibit limited generalization ability, increasing the likelihood of misclassification or low-confidence predictions. This reflects the model’s current challenges when dealing with long-tail distributions and open-set environments, which are common in real-world waste classification tasks. Moreover, although the LionBatch optimization strategy effectively reduces computational resource consumption and accelerates convergence, its performance is highly dependent on the quality and stability of training batches. In scenarios involving imbalanced data distributions or significant variation in sample features, LionBatch may result in slower convergence or increased performance fluctuations due to instability in the optimization process.

To address the existing limitations and further enhance the capabilities of HFWC-Net, future research will focus on several key directions. First, introducing more adaptive learning mechanisms will be a primary goal. These mechanisms could include techniques such as meta-learning, which allows the model to quickly adapt to new, unseen categories and improve generalization in dynamic environments. Self-supervised learning methods may also be explored to make the model more robust to variations in the dataset and improve its ability to generalize with minimal labeled data. Next, we aim to integrate advanced data augmentation strategies that will improve the model’s ability to handle rare and unseen categories. These strategies could include synthetic data generation through techniques like Generative Adversarial Networks (GANs), which can create realistic samples of rare or underrepresented waste types, thus enriching the training data. Mixup-based augmentation can also be used to improve the model’s ability to generalize by blending features from different classes. In terms of computational efficiency, we will explore parallel computing frameworks and distributed training architectures to optimize the training speed and resource utilization. Techniques like model pruning and quantization could be employed to reduce the computational burden and make the model more suitable for resource-constrained environments such as edge devices or mobile applications. By addressing these areas, we believe future work will enhance the versatility and practicality of HFWC-Net.

## 5. Conclusions

This study proposes a new Transformer-based framework called HFWC-Net. By integrating the local feature extraction capabilities of CNNs with the global contextual understanding of hybrid–modal visual Transformers, HFWC-Net leverages this CNN-Transformer synergy, enhanced by the innovative Agent Attention architecture. The use of agent tokens overcomes the limitations of traditional triple attention, enhancing the model’s flexibility, adaptability, and information capture capabilities, thereby improving training efficiency. Additionally, HFWC-Net employs the unbiased dynamic selection and adaptive adjustment pruning method, LionBatch, which dynamically reduces the number of samples in each training iteration. This approach improves computational efficiency, significantly speeds up model training, and optimizes the utilization of computing resources.

However, challenges remain, such as limited performance on low-resolution images and unseen categories, highlighting constraints in feature extraction and generalization. Future work will focus on developing adaptive learning techniques and robust preprocessing methods to improve performance in complex scenarios. Additionally, efforts will be directed toward optimizing the model for resource-constrained environments. Overall, HFWC-Net offers a powerful and efficient solution for waste classification, with the potential to advance environmental sustainability and improve urban waste management practices.

## Figures and Tables

**Table 1:** Categories and Sample Counts of the MixTrash Dataset.

Category	Subcategory	Quantity	Subcategory	Quantity	Subcategory	Quantity
Recyclable Waste	anvil	432	iron	83	scissors	317
	ashtray	137	kettle	184	scoop	174
	bag	463	keyboard	85	seasoningbottle	436
	book	756	knapsack	93	shampoobottle	417
	cage	322	milkbox	135	shoes	621
	cap	103	mobilephone	538	sodacan	687
	carton	586	mouse	339	stapler	385
	chair	66	oldscale	402	steelball	423
	chopsticks	371	oldclothes	407	table	138
	cigarettecase	611	paperbags	183	teapot	147
	comb	186	papercup	579	tincan	525
	cosmeticbottles	414	patchpanel	779	toys	666
	counter	702	pillow	382	trousers	496
	desklamp	68	pingpangracket	275	trunk	363
	earphone	728	plasticbag	424	tshirt	564
	electricfan	536	plasticbottle	654	tweezers	139
	electrickettle	174	plasticbowl	417	tyrepump	114
	eyeglass	93	plastichanger	442	usedtires	337
	filepocket	501	plushtoys	703	vacuumcup	144
	foambox	271	pot	466	vat	302
	gasstove	288	quilt	97	wasterpaper	567
	glassbottles	645	razor	315	watch	98
	glasscup	561	remotecontrol	536	woodenshovel	156
	hairdrier	93	router	199		
	hairstick	116	ruler	256		
Food Waste	applecore	679	eggshell	401	rice	559
	bananapeel	622	fishbone	419	snack	123
	bingtanghulu	133	frenchfries	76	vegetableleaf	558
	biscuit	488	sausage	807	wastebone	379
	bread	160	icecream	449	wastemeal	417
	cake	259	pulp	433	watermelonpeel	517
	chinese_vermicelli	67	residueoftea	696	wiltedflowers	109
Hazardous Waste	battery	663	glue	222	mosquitoswatter	295
	batterybutton	706	iccard	75	nail	177
	bulb	587	insecticide	352	ointment	401
	circuitboard	299	thermometer	553	powerbank	402
	capsule	709	modulatortube	588	pregnancykit	661
Residual Waste	adhesivetape	853	knittingwool	82	stickynote	142
	bandaid	87	lighter	440	strawhat	210
	ceramics	485	lunchbox	669	toothbrush	481
	cigarettebutt	614	mask	682	toothpick	135
	cottonswab	595	oldgloves	352	toothpick	135
	desiccant	533	pencil	524	towel	148
	featherduster	286	poker	148	umbrella	754
	firehydrant	282	socks	858	wetwipes	193
	flowerpot	412	sponge	168		

**Table 2:** Machine 1 Basic Properties.

Hardware	Device Information
Video memory	24 GB
Memory	128 GB
Hard disk	1 TB
Processor	Montage Jintide(R) C6230R
Graphics processor	NVIDIA GeForce GTX 3090
Operating system	Windows11 Python 3.9 Pytorch 2.1

**Table 3:** Machine 2 Basic Properties.

Hardware	Device Information
Video memory	12 GB
Memory	64 GB
Hard disk	1 TB
Processor	Intel(R) Core(TM) i7-12800F CPU
Graphics processor	NVIDIA GeForce GTX 3080Ti
Operating system	Ubuntu 20.04 LTS Python 3.8 Pytorch 2.0

**Table 4:** Comparison of the results of the proposed method and other classification methods on the Garbage Classification.

Method	Top1-Acc (%)	Precision (%)	Recall (%)	F1-Score (%)	Params (M)
VGGNet	93.97	93.81	93.84	93.79	134.26
GoogleNet	92.89	93.32	93.32	93.26	21.80
ResNet	93.29	93.65	93.61	93.58	23.50
DenseNet	93.52	93.66	93.58	93.52	12.33
MobileNet	94.74	94.63	94.65	94.59	4.32
EfficientNet	96.71	96.51	96.48	96.47	17.55
ConvNext	96.74	96.42	96.35	96.36	49.52
ViT	94.97	94.90	94.90	94.87	22.08
DeiT	94.87	95.11	95.06	95.02	21.71
Swin Transformer	96.12	96.23	96.19	96.18	48.94
CSwin Transformer	97.03	97.13	97.08	97.05	45.27
TNT	95.45	95.38	95.32	95.30	23.42
PiT	95.29	95.55	95.52	95.48	22.96
CaiT	96.32	96.17	96.10	96.09	46.58
BiFormer	96.45	96.43	96.39	96.38	25.09
VMamba	98.45	98.41	98.36	98.33	49.98
HFWC-Net (ours)	98.89	98.93	98.87	98.82	43.18

**Table 5:** Comparison of the results of the proposed method and other classification methods on the TrashNet.

Method	Top1-Acc (%)	Precision (%)	Recall (%)	F1 Score (%)	Params (M)
VGGNet	88.27	88.15	88.07	87.94	134.26
GoogleNet	83.50	83.61	82.90	82.50	21.80
ResNet	86.48	85.69	84.89	84.92	23.50
DenseNet	85.09	84.50	83.50	83.29	12.33
MobileNet	86.68	86.98	85.88	85.58	4.32
EfficientNet	88.87	88.59	88.27	88.07	17.55
ConvNext	91.85	91.13	91.05	90.94	49.52
ViT	90.06	89.88	89.66	89.62	22.08
DeiT	88.87	86.71	86.68	86.61	21.71
Swin Transformer	89.66	88.46	88.47	88.32	48.94
CSwin Transformer	92.36	92.17	92.24	92.08	45.27
TNT	90.26	89.93	89.66	89.56	23.42
PiT	88.47	88.90	88.87	88.86	22.96
CaiT	88.67	88.06	86.28	86.66	46.58
BiFormer	90.26	89.95	90.06	89.96	25.09
VMamba	95.83	95.78	95.57	95.48	49.98
HFWC-Net (ours)	96.43	96.44	96.39	96.32	43.18

**Table 6:** Comparison of the results of the proposed method and other classification methods on the MixTrash.

Method	Top1-Acc (%)	Precision (%)	Recall (%)	F1 Score (%)	Params (M)	Training Time per Epoch
VGGNet	72.11	71.13	70.66	69.97	134.26	02:22
GoogleNet	77.65	78.10	76.63	76.40	21.80	01:38
ResNet	80.30	80.25	79.36	78.85	23.50	01:36
DenseNet	80.59	81.11	80.12	79.76	12.33	02:21
MobileNet	82.37	81.95	81.35	80.92	4.32	01:37
EfficientNet	87.30	87.46	87.19	87.00	17.55	01:59
ConvNext	88.26	88.79	88.20	87.86	49.52	03:07
ViT	83.72	83.25	83.17	82.83	22.08	01:37
DeiT	83.56	82.27	82.33	81.92	21.71	02:11
Swin Transformer	85.32	86.05	85.51	85.09	48.94	02:38
CSwin Transformer	92.50	92.74	92.37	92.19	45.27	04:28
TNT	83.64	84.22	83.36	83.11	23.42	02:46
PiT	84.48	83.88	83.85	83.54	22.96	01:37
CaiT	85.78	86.83	85.72	85.32	46.58	03:31
BiFormer	87.28	87.83	87.24	87.12	25.09	04:50
VMamba	93.89	93.92	93.75	93.27	49.98	12:12
HFWC-Net (ours)	94.35	94.48	94.22	93.92	43.18	03:53

**Table 7:** Ablation of the results of the proposed method on the MixTrash.

Model	Agent Attention	Lion	InfoBatch	Top1-Acc (%)	Parmas (M)	Flops (G)	Total Training Time (h)
A				92.50	45.27	13.2	24.67
B	**√**			93.53	43.18	12.9	24.05
C		**√**		94.04	45.27	13.2	24.97
D			**√**	93.52	45.27	13.2	23.74
E	**√**	**√**		94.13	43.18	12.9	24.25
F		**√**	**√**	93.99	45.27	13.2	24.08
G	**√**		**√**	93.55	43.18	12.9	23.93
H	**√**	**√**	**√**	94.35	43.18	12.9	24.22

**Table 8:** GPU Memory Consumption and Inference Time of Models on MixTrash Dataset (Single Image).

Model	GPU Memory (M)	Average Inference Time (ms)
ResNet	279.1	4.2
EfficientNet	206.7	7.5
ConvNext	569.8	5.4
ViT	249.5	3.1
Swin Transformer	568.3	7.6
CSwin Transformer	263.4	8.3
BiFormer	290.0	14.5
HFWC-Net (ours)	266.2	7.9

## Data Availability

The datasets and source code used in this study are available upon reasonable request.
